# Elective Cesarean Section during Preterm Prevents Pulmonary Hypoplasia Development in Potter Sequence

**DOI:** 10.1155/2023/3216232

**Published:** 2023-01-31

**Authors:** Yuya Kinoshita, Rieko Sakamoto, Yusuke Hattori, Keishiro Furuie, Shohei Kuraoka, Yuko Hidaka, Hiroshi Tamura, Hiroshi Mitsubuchi, Kimitoshi Nakamura

**Affiliations:** Department of Pediatrics, Graduate School of Medicine Sciences, Kumamoto University, Kumamoto, Japan

## Abstract

Potter syndrome, first reported in 1946 by Edith Potter, refers to fatal cases of bilateral renal aplasia with pulmonary hypoplasia, peculiar facial features, and limb deformities. Presently, patients with oligohydramnios showing similar pathological manifestations due to oligohydramnios caused by conditions other than bilateral renal aplasia have been reported, and are known as the Potter sequence. There are limited studies and unclear guidelines on the safest delivery time and detailed postpartum management for patients with the Potter sequence. We experienced a case of Potter sequence, in which the patient was born by elective cesarean section at gestational age (GA) of 34 weeks. Fetal ultrasound at GA of 26 weeks 4 days showed oligohydramnios, multilocular cystic lesions in the left kidney, and an absent right kidney. Prenatal fetal MRI at GA of 33 weeks and 3 days showed pulmonary hypoplasia, and the ratio of fetal lung volume (FLV) to fetal body weight (FBW) was 0.0135 ml/g. We suspected that the fetal lung could not grow because of persistent oligohydramnios, which leads to a further decline in the ratio of FLV to FBW during pregnancy. We performed a cesarean section at GA of 34 weeks to prevent the exacerbation of the imbalance between lung volume and physique. We struggled to keep her condition stabilized with strict management of her respiratory condition, dialysis, and nutrition. She was discharged from the hospital at 169 days of age. Elective caesarean section in the term of premature birth prevented the progression of pulmonary hypoplasia and made it possible to save her life. Potter sequence is still relatively unknown, and it is necessary for more studies to be conducted in the future.

## 1. Introduction

Potter syndrome was first reported in 1946 by Edith Potter and refers to fatal cases of bilateral renal aplasia with oligohydramnios, resulting in pulmonary hypoplasia, peculiar facial features, and limb deformities [1]. At present, many cases of oligohydramnios showing similar pathological conditions without the underlying cause of bilateral renal aplasia have been reported and are known as the Potter sequence [2]. Potter sequence can be due to several reasons, with the primary cause being a reduction in amniotic fluid production. For example, oligohydramnios can occur due to water rupture for 2 weeks or more after the second trimester of pregnancy [3].

The incidence of the Potter sequence is 1 in 4000–6000 in Europe and the United States, and the male-female ratio ranges from 2 : 1 to 3 : 1, indicating a significantly higher occurrence in boys [3]. Fetal diagnosis of Potter sequence involves the detection of oligohydramnios and decreased lung volume by fetal ultrasonography and fetal magnetic resonance imaging (MRI). However, amniotic fluid production is not of renal origin until 12 weeks of fetal age, and therefore it is impossible to diagnose the Potter sequence prior to that age [3]. The survival rate of children with Potter sequence depends on the severity of pulmonary hypoplasia and, if necessary, subsequent control measures of the respiratory condition and renal dysfunction [4]. The overall prognosis is extremely poor for pulmonary hypoplasia due to the absence of an established and effective treatment [5, 6]. However, in recent years, a wide spectrum of the pathophysiology associated with the Potter sequence has been recognized, and there are some reports of long-term survival through intensive care, such as respiratory management (e.g., nitric oxide inhalation therapy) and dialysis therapy (e.g., continuous hemodialysis) [7, 8].

There are no reports regarding the safest delivery time and detailed postpartum management for patients with the Potter sequence. Considering this absence in literature, we report the case of a patient with the Potter sequence who underwent a cesarean section at gestational age (GA) of 34 weeks, in which we managed to stabilize the patient with strict management of the respiratory condition, dialysis, and proper nutrition.

## 2. Case Presentation

A 30-year-old woman underwent a natural pregnancy (second gravida). At a GA of 23 weeks and 4 days, the fetal bilateral kidneys could not be detected by fetal ultrasonography. At a GA of 26 weeks and 4 days, fetal ultrasound showed oligohydramnios (amniotic fluid index: 2 cm), unilateral kidney agenesis, and renal multilocular cystic lesions. Hence, she was referred to the obstetrics department in our institute for prenatal diagnosis and treatment. On the patient's first visit (GA of 26 weeks and 6 days), oligohydramnios was still observed. At a GA of 33 weeks and 3 days, the ratio of fetal lung volume (FLV) to fetal body weight (FBW) was 0.0135 mL/g. FLV was determined using an MRI, while FBW was determined using ultrasonography. Although the values for FLV/FBW indicating pulmonary hypoplasia are not clearly defined, data from Tanigaki et al. were used as a reference, in which FLV/FBW in patients with pulmonary hypoplasia and the control group were 0.012 mL/g ± 0.008 (mean ± SD) and 0.028 mL/g ± 0.007 (mean ± SD), respectively [9]. Owing to concerns about fetal pulmonary hypoplasia and its possible exacerbation from the continuation of pregnancy, we performed an elective caesarean section at a GA of 34 weeks and 2 days with the consent of the patient's parents, and a female baby was delivered. The patient weighed 1.9 kg with an Apgar score of 7 points at 1 min and 7 points at 5 min. She cried slightly at birth and could not maintain oxygen saturation within the normal range despite the administration of 100% oxygen. Tracheal intubation was performed immediately after birth to begin artificial respiration. After her admission to the neonatal intensive care unit, high-frequency oscillatory ventilation and the inhalation of nitric oxide were initiated, along with the administration of 100% oxygen. Surfactant administration was not an effective treatment method. Pulmonary hypoplasia was followed by persistent pulmonary hypertension and the arterial blood gas results from admission showed a partial pressure of arterial oxygen of 95.4 mmHg and an oxygenation index (mean airway pressure × fraction of inspired oxygen/partial pressure of oxygen) of 14.7, indicating extremely poor oxygenation. The patient had no peculiar facial features or limb deformities, and ultrasonography revealed multiple cysts in the left kidney and did not detect the right kidney. Chest radiograph at admission showed poor permeability in the lungs and a narrowed thorax, which was consistent with pulmonary hypoplasia. Therefore, the final diagnosis was Potter sequence with multicystic dysplastic kidney.

**Figure 1:**
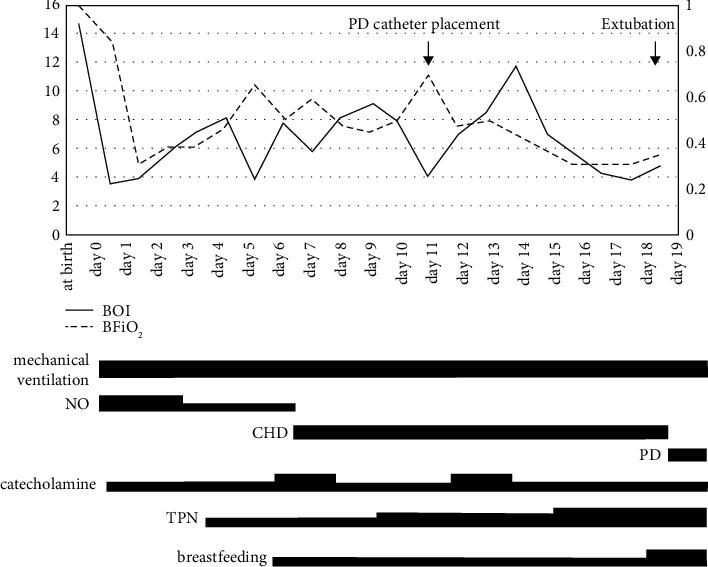
Clinical course immediately after birth. After birth, the patient underwent tracheal intubation and was mechanically ventilated. Systemic management with NO and catecholamines gradually improved OI and FiO_2_. At 5 days of age, NO was terminated, and CHD was started. Enteral nutrition started with breast milk at 4 days of age, but did not progress, so TPN gradually increased. PD catheter placement was performed at 11 days of age. At 18 days of age, CHD was switched to PD and extubated at 19 days of age. OI, oxygen index; PD, peritoneal dialysis; FiO_2_, fraction of inspired oxygen; NO, nitric oxide; CHD, continuous hemodialysis; TPN, total parenteral nutrition.

**Figure 2:**
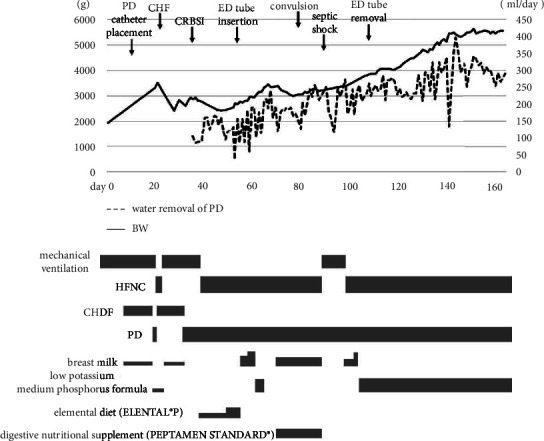
Clinical course from birth to discharge. The patient was extubated at 18 days of age and was reintubated at 24 days of age due to CHF-caused poor water removal. After water removal by PD became stable, she was extubated at 36 days of age and respiratory support was performed by HFNC. Elemental diet (ELENTAL® P) was also used in addition to breast milk and low potassium medium phosphorus formula, but enteral nutrition did not progress, so an ED tube was inserted at 53 days of age. Nutrition gradually increased and was aided by the high-protein digestive nutritional supplement (PEPTAMEN STANDARD®). After removing the ED tube, nutrition encompassed breast milk and later switched to the low potassium medium phosphorus formula. The preparation concentration of low potassium medium phosphorus formula gradually increased from 15% to 18%. During the course, complications such as CRBSI, septic shock, and convulsion occurred but were controlled by treatment. BW, body weight; PD, peritoneal dialysis; CHF, congestive heart failure; CRBSI, catheter-related blood stream infection; HFNC, high-flow nasal canula; ED, elemental diet; CHD, continuous hemodialysis.

The patient responded favorably to our treatment, and her best oxygen index (BOI) in the first 24 h improved from 14.7 to 3.5 (Figure 1). However, her respiratory condition gradually deteriorated due to atelectasis and excessive fluid accumulation in the lungs. Azotemia developed (serum creatine 4.97 mg/dL, blood urea nitrogen 35.2 mg/dL) due to nonfunctioning kidneys, and therefore, continuous hemodialysis (CHD) was started at 5 days of age. Administration of catecholamines was needed throughout the patient's hospitalization to stabilize the blood pressure during CHD. Catheter placement for peritoneal dialysis (PD) was performed at 11 days of age. We maintained the water valance and respiratory condition by CHD and PD with mechanical ventilation (Figure 1). CHD was discontinued after successful water removal by PD, which was confirmed at 32 days of age. The patient was extubated at 37 days of age as the respiratory condition stabilized and was switched to a high-flow nasal cannula. Enteral nutrition was provided from 4 days of age, and the patient was primarily managed by total parenteral nutrition (Figure 1). We started nutrition with breast milk and a low potassium medium phosphorus formula by nasal-gastric tube; however, enteral nutrition did not progress favorably, and intestinal dysfunction due to PD was suspected. Therefore, we attempted elemental diet therapy with ELENTAL® P using the elemental diet (ED) tube at 53 days of age. According to the report of Rees and Shaw, the target protein intake for infants undergoing PD is 2.3–3.0 g/kg/day [10]. However, it was difficult to reach the target protein amount under the strict control of water valance and electrolytes. Therefore, from 68 days of age, we changed the elemental diet to a digestive nutritional supplement (PEPTAMEN STANDARD®), which is higher in calories and protein than breast milk, along with the same water content as before. After starting this nutritional supplement, the patient gained weight despite abdominal distension (Figure 2). After removing the ED tube at 109 days of age, there were concerns about abdominal distension and vomiting. Therefore, the digestive nutritional supplement was discontinued, and a low potassium medium phosphorus formula was administered instead. To meet the protein requirements, the milk preparation concentration was gradually increased from 15% to 18%. After this increase, there were no instances of diarrhea, and the patient's weight gradually increased.

**Figure 3:**
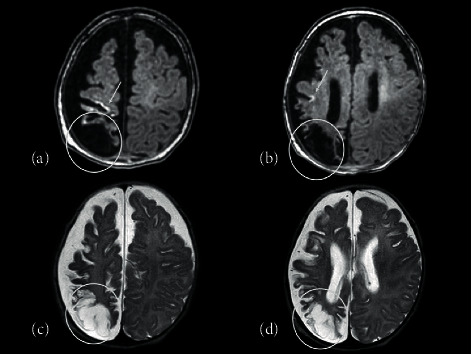
Head MRI. (a, b) T1-weighted MRI; (c, d) T2-weighted MRI. Parasagittal cerebral injury and ulegyria in the right parietal lobe and occipital lobe were observed. Arrows indicate parasagittal injury and circles indicate ulegyria.

Unfortunately, the patient experienced various complications during hospitalization. The complications were successfully resolved through the administration of antibiotics, anticonvulsants, and antihypertensive drugs (Figure 2). The brain MRI obtained on the 112^th^ day showed lesions in the right parietal and occipital lobes, suggesting the development of symptomatic epilepsy (Figure 3). She was discharged at 169 days of age. At the time of discharge, she was provided with a home-based high-flow nasal cannula, and enteral nutrition consisted of a combination of oral feeding and gastric tube administration. Moreover, at the time of discharge, she was able to hold her head up and smile. Currently, at the age of 1 year and 5 months, she is at about 5 to 6 months of developmental age, according to the Kyoto Scale of Psychological Development 2020. There were no irregularities in the developmental areas. She was able to roll over at 1 year of age; however, she still cannot sit up, and her growth rate is around −2SD for height and around 0 SD for weight. The home-based high-flow nasal cannula is no longer in use, oral feeding is slow, and gastric feeding continues.

## 3. Discussion

We encountered a case of Potter sequence born by elective caesarean section at a GA of 34 weeks and 2 days. In order to discuss delivery timing, it is necessary to consider the pathophysiology of the condition. The peculiar facial features and limb deformities, characteristic clinical features, occur because the fetus is pressed against the uterine wall due to oligohydramnios. The underlying cause for pulmonary hypoplasia, however, is unclear, with three possible hypotheses currently offered. The first is that the thorax is mechanically pressed against the uterine wall [11]. Second, the amniotic fluid itself is necessary for lung development, and therefore its insufficiency leads to pulmonary hypoplasia [12]. Third, the very presence of renal tissue in the first trimester is necessary for the early development of the lungs [13]. A characteristic of pulmonary hypoplasia is the low number of tracheal branches [13]. Tracheal branching of the fetal lung begins at GA of 12–16 weeks [14]. As amniotic fluid production mostly occurs in the placenta until GA of 12 weeks, its volume is maintained during the first trimester. Therefore, in addition to oligohydramnios, the reduction of tracheal branching might be caused by other factors. A functional kidney or its proline may be essential for tracheal branching development [12]. Glick et al. proposed that in congenital bilateral hydronephrosis, the time of delivery is determined by the presence or absence of oligohydramnios and the evaluation of renal function and lung maturity [15]. They suggested early delivery and, in some cases, termination as an option for patients with congenital bilateral hydronephrosis with oligohydramnios.

As per the results of several reports, it is reported that BOI within 24 hours of birth and the onset of oligohydramnios are prognostic factors of the Potter sequence. Mehler et al. reported that the survival rate of patients with renal oligohydramnios (ROH) is 58% and showed that a BOI < 9.6 within 24 hours of birth increased the chances of survival by almost 16-fold. They also showed that the onset of ROH beyond 28 weeks of gestation increased the chances of survival 50-fold [16]. Klaassen et al. reported that the survival rate of patients with ROH was 70% and, using the diagnostic median of 30 weeks as cutoff, diagnosis of ROH prior to 30 weeks gestation was associated with higher overall mortality than diagnosis after 30 weeks gestation [17]. In the present case, the patient was diagnosed with ROH at 26 weeks of gestation, and her prognosis was considered poor based on these reports. However, her BOI within 24 hours of birth was 3.5, which indicated a good prognosis. The fact that the patient was able to survive may suggest that the pulmonary hypoplasia at birth was not severe. Moreover, peculiar facial features and limb deformities could have appeared if the pregnancy had continued. Our case shows that early delivery might be a valid decision for preventing the exacerbation of the imbalance between lung volume and physique.

In addition to the delivery time regarding the Potter sequence, there is inadequate information on detailed management methods. There are reports that the prognosis of the Potter sequence has improved, but some of these cases retained kidney function and did not require mechanical ventilation and/or dialysis [16, 17]. Since the Potter sequence encompasses a spectrum of diseases, every case should not be subjected to the same treatment methods. Presently, there is little information on the prognosis and management of patients with severe pulmonary hypoplasia without renal function. In the present case, the patient's blood pressure instability was attributed to the inactivity of her renin-angiotensin-aldosterone system due to a lack of renal function. In addition to respiratory and dialysis management, nutritional management was challenging. It has long been reported that pediatric patients with renal failure often have low overall calorie intake due to insufficient protein intake [18]. Additionally, total calorie intake has a positive correlation with the growth rate in children undergoing PD [19]. Nutritional management of patients with PD is crucial if kidney transplantation is being considered in the future, and clinicians should note the difficulty in controlling water balance and electrolyte concentration. We believe that the administration of high-calorie (high-protein) nutritional supplements through ED tubes is useful for the management of lightweight cases with PD.

This study has some limitations. First, it is unknown what the outcome would have been if this case had been born at term, and there is no way to confirm if early intervention improved the prognosis. Second, although the nutritional management of this case was difficult, it is unclear whether such a course is common in low-birth-weight infants with PD. In the future, it will be necessary to collect data on PD in low-birth-weight infants.

We experienced a case of the Potter sequence with no renal function and severe pulmonary hypoplasia. There are many unclear aspects regarding the delivery time and management method of the Potter sequence. Therefore, we believe it is necessary to accumulate more information about the Potter sequence through future studies to develop effective management and treatment methods.
